# Five New Diterpenoids from an Okinawan Soft Coral, *Cespitularia* sp. 

**DOI:** 10.3390/md10122741

**Published:** 2012-11-30

**Authors:** Prodip K. Roy, Wilmar Maarisit, Michael C. Roy, Junsei Taira, Katsuhiro Ueda

**Affiliations:** 1 Department of Chemistry, Biology and Marine Science, University of the Ryukyus, Nishihara-cho, Okinawa 903-2013, Japan; Email: prodipkroy@gmail.com (P.K.R.); wmaarisit@yahoo.com (W.M.); 2 Biological Resources Section, Research Support Division, Okinawa Institute of Science and Technology, Okinawa 904-0495, Japan; Email: mcroy71@hotmail.com; 3 Department of Bioresource Technology, Okinawa National College of Technology, Nago-shi, Okinawa 905-2192, Japan; Email: taira@okinawa-ct.ac.jp

**Keywords:** *Cespitularia*, cytotoxicity, diterpenoid, HCT 116 cells, alcyonolide

## Abstract

Five new diterpenoids **1**–**5** were isolated from an Okinawan soft coral, *Cespitularia* sp., together with the known diterpenoid, alcyonolide (**6**). New diterpenoid structures were elucidated by spectroscopic methods and by comparison of their NMR data with those of related compounds. Alcyonolide (**6**) was cytotoxic against HCT 116 cells (IC_50_ 5.85 μM), while these new diterpenoids **1**–**5** were much less active (IC_50_ 28.2–91.4 μM).

## 1. Introduction

**Figure 1:**
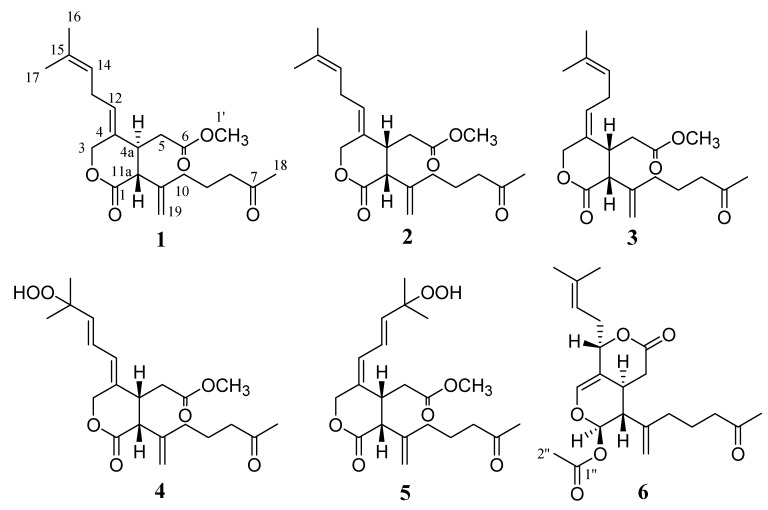
Structures of compounds **1**–**6**.

Soft corals are rich sources of structurally unique and biologically active metabolites [1,2]. As part of our continuous search for bioactive secondary metabolites from Okinawan marine organisms [3,4,5], we isolated and characterized five new diterpenoids **1**–**5** as well as the known alcyonolide (**6**) [6] from a soft coral, *Cespitularia* sp. (Figure 1). Alcyonolide was the major constituent of ethyl acetate extracts. The carbon skeleton of **1**–**6** corresponds to a seco-type variety of xenicins, possessing a nine-membered carbocyclic ring *trans*-fused to a dihydropyran ring [7,8,9]. The biogenesis of compounds **1**–**6** presumably proceeds after completion of the xenicin-type carbon framework [7,8,9]. Herein, we report the isolation, structure elucidation, and cytotoxicity of the isolates from the soft corals. 

## 2. Results and Discussion

### Structure Analysis and Characterization of Compounds ***1–6***

The soft coral *Cespitularia* sp. was collected from the coast of Zamami Island, Okinawa, and extracted with acetone. The EtOAc-soluble portion of the acetone extract inhibited 80% of the first cleavage of fertilized sea urchin eggs at 20 μg/mL. Fractionation of the toxic extract by silica gel column chromatography followed by normal phase HPLC purification gave six compounds: [**1** (0.0043%, wet weight), **2** (0.0135%), **3** (0.0026%), **4** (0.0017%), **5** (0.0012%)] and alcyonolide (**6**) (0.26%). Alcyonolide (**6**) was unambiguously identified by comparison of its spectral data with those described in the literature [6,10].

**Figure 2:**
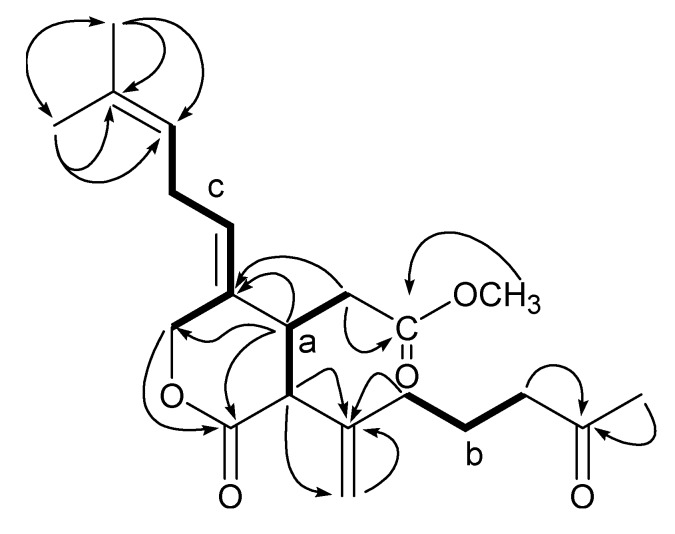
Partial structures (**a**, **b** and **c**) of **1** based on COSY (bold line) and key HMBC correlations (arrows H/C).

The high resolution nanospray-ionization MS (HRNSIMS) of **1** showed a pseudomolecular ion peak [M + H]^+^ at *m/z* 363.2168 (calcd. for C_21_H_31_O_5_, 363.2166). IR absorption bands at 1734 and 1714 cm^−1^ indicated the presence of several carbonyl groups. ^1^H and ^13^C NMR data (Table 1 and Table 2) of **1 **suggested that it was a diterpene derivative. NMR data of **1** indicated the presence of one ketone (δ_C_ 208.5), two esters (δ_C_ 172.4 and 170.9), two trisubstituted double bonds [δ_C_ 133.5, 129.0, 128.5 (δ_H_ 5.46 t, *J *= 7.4 Hz) and 120.9 (δ_H_ 4.98 br t, *J *= 7.0 Hz)], one terminal methylene [δ_C_ 143.0 and δ_C_ 115.6 (δ_H_ 5.13 s and 5.09 s)], two methines [δ_C_ 56.1 (δ_H_ 3.10 d, *J *= 11.6 Hz) and δ_C_ 39.7 (δ_H_ 3.12 ddd, *J *= 11.6, 5.2, 4.3 Hz)], five methylenes [δ_C_ 43.0 (δ_H_ 2.43 t, *J *= 7.3 Hz), δ_C_ 34.1 (δ_H_ 2.02 m), δ_C_ 34.0 (δ_H_ 2.55 dd, *J *= 16.7, 4.3 Hz and 2.58 dd, *J *= 16.7, 5.2 Hz), δ_C_ 26.8 (δ_H_ 2.64 m) and δ_C_ 21.5 (δ_H_ 1.71 m)] and four methyls [δ_C_ 52.0 (δ_H_ 3.61 s), δ_C_ 30.1 (δ_H_ 2.13 s), δ_C_ 25.8 (δ_H_ 1.68 s) and δ_C_ 17.9 (δ_H_ 1.60 s)]. Among the four methyls, one was associated with the ketonic carbonyl (HMBC correlations of H_3_-18/C-7, -8), another was assigned to the methyl ester (HMBC correlation of H_3_-1′/C-6), and the remaining methyls were part of an isobutenyl group (HMBC correlations of H_3_-16/C-14, -15, -17 and H_3_-17/C-14–C-16). Comparison of NMR data of **1 **and **6 **revealed similarities. However, there were several significant differences that indicated the presence of new functional groups in **1**. The major difference was the presence of a methyl ester and the absence of an acetal group, an acetyl group and an oxygenated methine in **1**. Three major spin systems were constructed on the basis of COSY correlations, as shown in Figure 2 [H-11a/H-4a/H-5 for spin system **a**, H-8/H-9/H-10 for spin system **b** and H-12/H-13/H-14 and H-3/H-12 (a long range coupling) for spin system **c**]. The partial structures (**a**, **b** and **c**) and other fragments (C-1, C-6–C-1′, C-11–C-19, C-7–C-18 and C-15–C-17) were connected by HMBC correlations (H-3/C-1; H-4a/C-1, -3, -4; H-1′, -5/C-6; H-10/C-11; H-11a/C-11, -19; H-8, -18/C-7; H-16/C-14, -15, -17; H-17/C-14–C-16). Thus, the planner structure was established as shown in Figure 2.

**Table 1:** ^1^H NMR data (CDCl_3_, 500 MHz) for compounds **1**–**6**.

δ_H_ (mult., *J* in Hz)						
H No.	**1**	**2**	**3**	**4**	**5**	**6**
1						5.94 (d, 7.5)
3	4.91 (s)	4.89 (m)	4.60 (d, 12.9)	5.03 (s)	4.94 (d, 14.0)	6.38 (s)
			4.87 (d, 12.9)		4.70 (d, 14.0)	
4a	3.12 (ddd, 11.6, 5.2, 4.3)	3.12 (br q, 6.4)	3.34 (br q, 6.1)	3.22 (br q, 6.2)	3.69 (br q, 6.0)	2.72 (m)
5	2.55 (dd, 16.7, 4.3)	2.51 (dd, 16.1, 6.9)	2.54 (dd, 16.3, 7.6)	2.55 (dd, 16.5, 6.6)	2.57 (dd, 16.0, 5.9)	2.29 (dd, 18.6, 12.5)
	2.58 (dd, 16.7, 5.2)	2.56 (dd, 16.1, 6.5)	2.56 (dd, 16.3, 4.7)	2.60 (dd, 16.5, 6.5)	2.62 (dd, 16.0, 6.9)	2.76 (dd, 18.6, 6.9)
8	2.43 (t, 7.3)	2.47 (m)	2.48 (m)	2.48 (m)	2.50 (m)	2.44 (t, 7.0)
9	1.71 (m)	1.81 (m)	1.79 (m)	1.78 (m)	1.80 (m)	1.73 (q, 7.0)
10	2.02 (m)	2.08 (m)	2.09 (m)	2.08 (m)	2.10 (m)	1.79 (m)
					2.02 (m)	
11a	3.10 (d, 11.6)	3.38 (d, 6.4)	3.51 (d, 6.1)	3.43 (d, 6.2)	3.50 (d, 6.0)	2.20 (t, 8.0)
12	5.46 (t, 7.4)	5.41 (tq, 7.4, 1.6)	5.50 (t, 7.0)	6.07 (d, 11.0)	6.10 (d, 11.1)	4.75 (t, 7.5)
13	2.64 (m)	2.65 (m)	2.75 (m)	6.21 (dd, 15.3, 11.0)	6.53(dd, 15.4, 11.1)	2.43 (m)
						2.52 (m)
14	4.98 (br t, 7.0)	5.00 (br t, 7.0)	5.03 (br t, 7.0)	5.85 (d, 15.3)	5.85 (d, 15.4)	5.80 (t, 7.5)
16	1.60 (s)	1.60 (s)	1.63 (s)	1.36 (s)	1.36 (s)	1.61 (s)
17	1.68 (s)	1.69 (s)	1.70 (s)	1.56 (s)	1.55 (s)	1.70 (s)
18	2.13 (s)	2.14 (s)	2.13 (s)	2.14 (s)	2.14 (s)	2.12 (s)
19a	5.09 (s)	4.94 (s)	4.95 (s)	4.95 (s)	4.96 (s)	4.91 (s)
19b	5.13 (s)	5.07 (s)	5.07 (s)	5.08 (s)	5.08 (s)	5.01 (s)
1′	3.61 (s)	3.66 (s)	3.65 (s)	3.68 (s)	3.66 (s)	
2″						2.08 (s)

**Table 2:** ^13^C NMR data (CDCl_3_, 125 MHz) for compounds **1**–**6**.

δc						
C No.	**1**	**2**	**3**	**4**	**5**	**6**
1	170.9 (C)	171.8 (C)	171.8 (C)	171.4 (C)	172.0 (C)	92.9 (CH)
3	66.8 (CH_2_)	66.7 (CH_2_)	72.0 (CH_2_)	66.7 (CH_2_)	71.5 (CH_2_)	137.7 (CH)
4	129.0 (C)	129.8 (C)	130.5 (C)	132.2 (C)	132.2 (C)	110.4 (C)
4a	39.7 (CH)	38.2 (CH)	33.5 (CH)	38.3 (CH)	33.7 (CH)	31.1 (CH)
5	34.0 (CH_2_)	38.9 (CH_2_)	37.8 (CH_2_)	38.8 (CH_2_)	38.6 (CH_2_)	34.9 (CH_2_)
6	172.4 (C)	171.9 (C)	172.0 (C)	171.7 (C)	171.8 (C)	169.9 (C)
7	208.5 (C)	208.7 (C)	208.8 (C)	208.8 (C)	210.0 (C)	208.1 (C)
8	43.0 (CH_2_)	42.9 (CH_2_)	42.9 (CH_2_)	42.9 (CH_2_)	42.8 (CH_2_)	42.9 (CH_2_)
9	21.5 (CH_2_)	21.5 (CH_2_)	21.6 (CH_2_)	21.5 (CH_2_)	21.9 (CH_2_)	21.5 (CH_2_)
10	34.1 (CH_2_)	33.0 (CH_2_)	33.3 (CH_2_)	33.0 (CH_2_)	33.2 (CH_2_)	34.9 (CH_2_)
11	143.0 (C)	143.3 (C)	143.4 (C)	143.0 (C)	143.1 (C)	145.1 (C)
11a	56.1 (CH)	52.4 (CH)	51.9 (CH)	52.2 (CH)	52.3 (CH)	48.8 (CH)
12	128.5 (CH)	127.9 (CH)	129.6 (CH)	127.0 (CH)	128.0 (CH)	79.5 (CH)
13	26.8 (CH_2_)	26.7 (CH_2_)	27.1 (CH_2_)	123.7 (CH)	123.6 (CH)	35.1 (CH_2_)
14	120.9 (CH)	121.0 (CH)	120.8 (CH)	139.9 (CH)	141.1 (CH)	117.9 (CH)
15	133.5 (C)	133.5 (C)	133.7 (C)	82.3 (C)	82.2 (C)	136.0 (C)
16	17.9 (CH_3_)	17.9 (CH_3_)	18.0 (CH3)	24.4 (CH_3_)	25.1 (CH_3_)	18.2 (CH_3_)
17	25.8 (CH_3_)	25.2 (CH_3_)	25.8 (CH3)	24.5 (CH_3_)	24.4 (CH_3_)	25.9 (CH_3_)
18	30.1 (CH_3_)	30.1 (CH_3_)	30.1 (CH3)	30.2 (CH_3_)	30.2 (CH_3_)	30.1 (CH_3_)
19	115.6 (CH_2_)	114.5 (CH_2_)	114.5 (CH2)	114.7 (CH_2_)	115.0 (CH_2_)	113.7 (CH_2_)
1′	52.0 (CH_3_)	51.9 (CH_3_)	52.0 (CH3)	52.1 (CH_3_)	52.2 (CH_3_)	
1″						169.3 (C)
2″						20.9 (CH_3_)

The ∆^4,12^ configuration was assigned as *Z* on the basis of NOEs (H-3/H-13 and H-4a/H-12). Since overlap of the H-4a and H-11a proton signals in CDCl_3_ prevented us from determining the configuration of the ring junction at C-4a/C-11a, NMR spectra were recorded in C_6_D_6_ and clearly separated signals were observed for the H-4a (δ_H_ 2.96) and H-11a (δ_H_ 3.12) (Experimental Section). Irradiation of these signals did not show any NOEs, suggesting their *trans* orientation as in alcyonolide (**6**). This was further supported by a large coupling constant (*J*_H4a,11a_ = 11.6 Hz) [7,8,9]. Compound **1** could be a precursor of alcyonolide (**6**) [10], and assuming a common biosynthetic route for them, the absolute configuration could be as depicted in Figure 1.

Compound **2 **had the same molecular formula as **1**, as deduced from HRNSIMS [*m/z* 363.2166 (M + H)^+^, calcd. for C_21_H_31_O_5_, 363.2166]. IR absorption bands at 1738 and 1725 cm^−1^ indicated the presence of several carbonyl groups. ^1^H and ^13^C NMR data (Table 1 and Table 2) of **2** are very similar to those of **1**, except for NMR resonances of H-4a, C-4a, H-11a and C-11a, suggesting that **1** and **2** could be *cis*/*trans* isomers. Extensive analysis of 1D and 2D NMR data led to a planar structure of **2**, which places it in the same diterpene class as **1**. Geometric configuration of the ring junction at C-4a/C-11a in **2** was assigned to be *cis* by NOEDS experiments, in which irradiation of H-11a (δ_H_ 3.38 d, *J* = 6.4 Hz) caused enhancement of H-4a (δ_H_ 3.12 br q, *J* = 6.4 Hz). NOEs observed between H-3/H-13 and H-4a/H-12 revealed *Z* configuration of the ∆^4,12^, as in **1**.

Compound **3 **also had the same molecular formula as **1 **and **2**, as deduced from HRNSIMS [*m/z* 363.2167 (M + H)^+^, calcd. for C_21_H_31_O_5_, 363.2166]. IR absorption bands at 1730 and 1709 cm^−1^ indicated the presence of several carbonyl groups. Its ^1^H and ^13^C NMR data (Table 1 and Table 2) also showed similarities to those of **1** and **2**. Extensive analysis of 1D and 2D NMR data and comparison of the ^1^H and ^13^C NMR data with those of **2** led to the same planar structure as **2**, except for geometry of the double bond at C-4. In contrast to **2**, the ∆^4,12^ was assigned as *E *configuration in **3** based on NOEs (H-3/H-12 and H-4a/H-13). The *cis* ring junction at C-4a/C-11a was established on the basis of an NOE between H-4a (δ_H_ 3.34 br q, *J* = 6.1 Hz) and H-11a (δ_H_ 3.51 d, *J* = 6.1 Hz) as in **2**. 

The HRNSIMS of **4** showed a pseudomolecular ion peak [M + Na]^+^ at *m/z* 417.1891 (calcd. for C_21_H_30_O_7_Na, 417.1884). The molecular formula of **4** differed from those of **1**–**3** by the addition of two oxygens. IR absorption bands at 1742 and 1729 cm^−1^ also indicated the presence of several carbonyl groups as in **1**–**3**. Comparison of NMR data (Table 1 and Table 2) showed similarities between **2** and **4**. However, there were several significant differences that indicated the presence of a new functional group in **4**. In the ^1^H NMR spectrum, two methyl signals at δ_H_ 1.60 (s) and δ_H_ 1.69 (s), assigned in **2** as vinyl methyls at the terminal carbon, were shifted upfield to δ_H_ 1.36 (s) and δ_H_ 1.56 (s), respectively, in **4**. In addition, NMR data revealed the presence of a *trans* double bond [δ_C_ 123.7, δ_H_ 6.21 (dd, *J* = 15.3, 11.0 Hz); δ_C_ 139.9, 5.85 (d, *J* = 15.3 Hz)] and an oxygenated quaternary carbon [δ_C_ 82.3 (s)]. These changes are accommodated well by the migration of the C-14, -15 double bond to the C-13, -14 position. The oxygenated quaternary carbon (δ_C_ 82.3) was placed at C-15 on the basis of HMBC correlations (H-14, -16, -17/C-15). Extensive analysis of the 1D and 2D NMR data led to the planar structure of **4**, as shown in Figure 1. A positive iodine-starch test also supported the presence of the hydroperoxy group in **4** [11]. Geometric configuration of the ring junction at C-4a/C-11a in **4** was also assigned to be *cis* by NOEDS experiments, in which irradiation of H-11a (δ_H_ 3.43 d, *J* = 6.2 Hz) caused enhancement of H-4a (δ_H_ 3.22 br q, *J* = 6.2 Hz). NOEs observed between H-3/H-13 and H-4a/H-12 revealed *Z* configuration of the ∆^4,12^, as in **1** and **2**. Compound **4** could be formed by the ene reaction between **2** and a singlet oxygen. 

Compound **5** had the same molecular formula as **4**, as deduced from HRNSIMS [*m/z* 417.1891 (M + Na)^+^, calcd. for C_21_H_30_O_7_Na, 417.1884]. IR spectrum of **5** was almost identical to that of **4** indicating the presence of several carbonyl groups (1742 and 1729 cm^−1^). ^1^H and ^13^C NMR spectral data (Table 1 and Table 2) of **5** were also similar to those of **4**, except for NMR resonances of H-3, C-3, H-4a, C-4a and H-13. Extensive analysis of 1D and 2D NMR data and comparison of the ^1^H and ^13^C NMR data with those of **4** led to the same planar structure as **4**, except for geometry of the double bond at C-4. In contrast to **4**, the ∆^4,12^ was assigned as *E *configuration in **5** on the basis of NOEs (H-3/H-12 and H-4a/H-13). The NOE between H-4a (δ_H_ 3.69 br q, *J* = 6.0 Hz) and H-11a δ_H_ 3.50 d, *J* = 6.0 Hz) allowed the ring junction to be assigned as *cis*, as in **2**, **3** and **4**. Compound **5** could be also formed by the ene reaction between **3** and a singlet oxygen. 

Since these compounds were isolated from the cytotoxic EtOAc extract, compounds **1**–**6** were tested for cytotoxicity against HCT116 cells (human colorectal cancer cells). IC_50_ values of isolates **1**–**6** against HCT116 cells were 28.18, 91.35, 89.48, 39.24, 71.44 and 5.85 μM, respectively. 

## 3. Experimental Section

### 3.1. General Experimental Procedures

Optical rotation was measured using a JASCO P-1010 Polarimeter. UV spectra were obtained with a HITACHI U-2001 Spectrophotometer. NMR spectra were recorded on a Bruker AvanceIII 500 spectrometer in CDCl_3_ or C_6_D_6_. Chemical shifts and coupling constants were given as δ and Hz, respectively. IR spectra were recorded on a JASCO FT/IR-6100 Fourier Transform Infrared Spectrometer. High resolution mass spectra (HRMS) were obtained on an LTQ Orbitrap hybrid mass spectrometer equipped with a nanospray ionization (NSI) source. Open column chromatography was performed on Kieselgel 60 (70–230 mesh, Merck). HPLC was performed using a COSMOSIL Si60 HPLC column (5SL, 10 × 250 mm). Analytical TLC was performed using Kieselgel 60 F_254_ DC-fertigplatten (Merck). All solvents were reagent grade.

### 3.2. Animal Materials

The soft coral was collected during low tide from the coast of Zamami Island, Okinawa, Japan, in April 2012, and identified as *Cespitularia* sp. A voucher specimen was deposited at the University of the Ryukyus (Specimen No. 110312). 

### 3.3. Extraction and Compounds Isolation

Samples (2.9 kg, wet weight) of *Cespitularia* sp. overgrown on a coral reef were collected by hand, transported to lab and extracted with acetone (5 L × 2). After filtration, extracts were concentrated under reduced pressure to make an acetone extract. The acetone extract was partitioned between H_2_O (200 mL) and EtOAc (200 mL × 2). After evaporation of the solvent, the EtOAc fraction yielded a solid crude extract (30.24 g). The EtOAc extract inhibited 80% of the first cleavage of fertilized sea urchin eggs at 20 μg/mL. A portion of the crude extract (13.62 g) was first chromatographed on silica gel to give 11 fractions (Hexane/EtOAc gradient). The seventh fraction contained alcyonolide (**6**) (1.73 g). A part (309.7 mg) of the sixth fraction (2.5 g) was subjected to further purification by HPLC on a COSMOSIL Si60 column using hexane/EtOAc (1:1) to give eleven subfractions. Subfraction four yielded diterpenoid **1** (7.1 mg); subfraction five yielded alcyonolide (**6**) (214.6 mg); subfraction seven yielded diterpenoid **5** (2.0 mg); subfraction eight yielded diterpenoid **4** (2.8 mg). The second subfraction (49.7 mg) was purified by HPLC on a COSMOSIL Si60 column using hexane/EtOAc (3:1) to yield diterpenoid **2** (22.0 mg) and diterpenoid **3** (4.3 mg).

Compound **1**: Colorless oil; [α]_D_^26^ −7.27 (*c* 0.22 CHCl_3_); FT/IR ν_max_ (film) 3407, 2928, 2360, 2341, 1734, 1714, 1434, 1367 and 1163 cm^−1^; ^1^H and ^13^C NMR (CDCl_3_) data are listed in Table 1 and Table 2; ^1^H NMR (C_6_D_6_, 500 MHz) δ 5.39 (br t, *J = *7.4 Hz, H-12), 5.08 (s, H-19), 4.92 (s, H-19), 4.94 (br t, *J = *7.0 Hz, H-14), 4.52 (d, *J = *14.5 Hz, H-3), 4.39 (d, *J = *14.5 Hz, H-3), 3.27 (s, H_3_-1′), 3.12 (d, *J = *11.6 Hz, H-11a), 2.96 (m, H-4a), 2.17 (dd, *J = *16.3, 5.7 Hz, H-5), 2.39 (dd, *J = *16.3, 4.5 Hz, H-5), 2.34 (t, *J = *7.3 Hz, H-8), 2.01 (m, H-10), 1.89 (m, H-13), 1.63 (s, H_3_-18), 1.60 (m, H-9), 1.57 (s, H_3_-17), 1.41 (s, H_3_-16). ^13^C NMR (C_6_D_6_, 125 MHz) δ 206.2 (C-7), 172.5 (C-6), 169.8 (C-1), 144.1 (C-11), 133.1 (C-15), 130.5 (C-4), 128.2 (C-12), 121.9 (C-14), 115.5 (C-19), 66.4 (C-3), 56.8 (C-11a), 51.6 (C-1′), 29.6 (C-8), 40.4 (C-4a), 34.7 (C-10), 34.1 (C-5), 29.6 (C-18), 27.0 (C-13), 25.9 (C-17), 22.0 (C-9), 17.9 (C-16). HRNSIMS *m/z* [M + Na]^+^ 385.1988 (calcd. for C_21_H_31_O_5_Na_,_ 385.1985), [M + H]^+^ 363.2168 (calcd. for C_21_H_31_O_5_, 363.2166).

Compound **2**: Colorless oil; [α]_D_^26^ +3.33 (*c* 0.78 CHCl_3_); FT/IR ν_max_ (film) 2359, 2339, 1738, 1725, 1439, 1363 and 1164 cm^−1^; ^1^H and ^13^C NMR (CDCl_3_) data are listed in Table 1 and Table 2; HRNSIMS *m/z* [M + Na]^+^ 385.1993 (calcd. for C_21_H_30_O_5_Na, 385.1985), [M + H]^+^ 363.2166 (calcd. for C_21_H_31_O_5_, 363.2166).

Compound **3**: Colorless oil; [α]_D_^27^ +18.91 (c 0.37 CHCl_3_); FT/IR ν_max_ (film) 2360, 2341, 1730, 1709,1435, 1360 and 1160 cm^−1^; ^1^H and ^13^C NMR (CDCl_3_) data are listed in Table 1 and Table 2; HRNSIMS *m/z* [M + Na]^+^ 385.1986 (calcd. for C_21_H_30_O_5_Na, 385.1985), [M + H]^+^ 363.2167 (calcd. for C_21_H_31_O_5_, 363.2166).

Compound **4**: Colorless oil; [α]_D_^27^ +1.5 (*c* 0.13 CHCl_3_); FT/IR (film) ν_max_ 2359, 2343, 1742, 1729, 1367, 1240 and 1164 cm^−1^; UV λ_max_ 257 (log ε 3.9) nm; ^1^H and ^13^C NMR (CDCl_3_) data are listed in Table 1 and Table 2; HRNSIMS *m/z* [M + Na]^+^ 417.1891 (calcd. for C_21_H_30_O_7_Na, 417.1884), [M + K]^+^ 433.1631 (calcd. for C_21_H_30_O_7_K, 433.1623).

Compound **5**: Colorless oil; [α]_D_^27^ +6.15 (*c* 0.13 CHCl_3_); FT/IR (film) ν_max_ 2359, 2343, 1742, 1729, 1367, 1224 and 1160 cm^−1^; UV λ_max_ 257 (log ε 3.9) nm; ^1^H and ^13^C NMR (CDCl_3_) data are listed in Table 1 and Table 2; HRNSIMS *m/z* [M + Na]^+^ 417.1891 (calcd. for C_21_H_30_O_7_Na, 417.1884), [M + K]^+^ 433.1631 (calcd. for C_21_H_30_O_7_K, 433.1623).

## 4. Conclusion

Five new diterpenoids **1**–**5**, and alcyonolide (**6**) were isolated from the soft coral *Cespitularia *sp. Alcyonolide was the major constituent of the ethyl acetate extract. Their structures were determined by spectroscopic methods. Alcyonolide showed IC_50_ values of 5.85 μM against HCT 116 cells, while diterpenoids **1**–**5** were active only at significantly higher dose (IC_50_ 28.2–91.4 μM). It is likely that the the lactone moiety (C-6–C-5–C-4a–C-4–C-12) and/or the acetal at C-1 are necessary for the cytotoxicity. Compounds **4** and **5** could be artifacts, produced by autoxidation of **2** and **3** during isolation process. Because of the abundance of alcyonolide, further chemical derivatizations and other bioassays are now being undertaken. 
